# GATA Factor Regulation in Excess Nitrogen Occurs Independently of Gtr-Ego Complex-Dependent TorC1 Activation

**DOI:** 10.1534/g3.115.019307

**Published:** 2015-05-29

**Authors:** Jennifer J. Tate, Isabelle Georis, Rajendra Rai, Fabienne Vierendeels, Evelyne Dubois, Terrance G. Cooper

**Affiliations:** * Department of Microbiology, Immunology and Biochemistry, University of Tennessee Health Science Center, Memphis, Tennessee 38163; † Institut de Recherches Microbiologiques J.-M. Wiame, Laboratoire de Microbiologie Université Libre de Bruxelles, Brussels B1070, Belgium

**Keywords:** Gln3 localization, Gtr1/2 complex, Ego1/3 complex, Tor complex one (TORC1), nitrogen catabolite repression (NCR), nitrogen limitation, nitrogen starvation

## Abstract

The TorC1 protein kinase complex is a central component in a eukaryotic cell’s response to varying nitrogen availability, with kinase activity being stimulated in nitrogen excess by increased intracellular leucine. This leucine-dependent TorC1 activation requires functional Gtr1/2 and Ego1/3 complexes. Rapamycin inhibition of TorC1 elicits nuclear localization of Gln3, a GATA-family transcription activator responsible for the expression of genes encoding proteins required to transport and degrade poor nitrogen sources, *e.g.*, proline. In nitrogen-replete conditions, Gln3 is cytoplasmic and Gln3-mediated transcription minimal, whereas in nitrogen limiting or starvation conditions, or after rapamycin treatment, Gln3 is nuclear and transcription greatly increased. Increasing evidence supports the idea that TorC1 activation may not be as central to nitrogen-responsive intracellular Gln3 localization as envisioned previously. To test this idea directly, we determined whether Gtr1/2- and Ego1/3-dependent TorC1 activation also was required for cytoplasmic Gln3 sequestration and repressed GATA factor-mediated transcription by abolishing the Gtr-Ego complex proteins. We show that Gln3 is sequestered in the cytoplasm of *gtr1*Δ, *gtr2*Δ, *ego1*Δ, and *ego3*Δ strains either long term in logarithmically glutamine-grown cells or short term after refeeding glutamine to nitrogen-limited or -starved cells; GATA factor−dependent transcription also was minimal. However, in all but a *gtr1*Δ, nuclear Gln3 localization in response to nitrogen limitation or starvation was adversely affected. Our data demonstrate: (i) Gtr-Ego-dependent TorC1 activation is not required for cytoplasmic Gln3 sequestration in nitrogen-rich conditions; (ii) a novel Gtr-Ego-TorC1 activation-independent mechanism sequesters Gln3 in the cytoplasm; (iii) Gtr and Ego complex proteins participate in nuclear Gln3-Myc^13^ localization, heretofore unrecognized functions for these proteins; and (iv) the importance of searching for new mechanisms associated with TorC1 activation and/or the regulation of Gln3 localization/function in response to changes in the cells’ nitrogen environment.

The well-being and reproductive fitness of all living cells depends on the availability of a sufficient nitrogen supply, ranging from atmospheric nitrogen in the case of plants and some micro-organisms to reduced and/or organo-nitrogenous compounds such as urea, amino acids, etc. in the case of lower and higher eukaryotic cells. Mechanisms whereby eukaryotic cells are able to maintain homeostasis by quickly and effectively responding to varying nitrogen availability has been a field of intense investigation. The Target of Rapamycin Complex 1 (TorC1) is one of two Tor containing complexes that play a central role in these responses (Loewith *et al.* 2002; Reinke *et al.* 2004). At the center of TorC1 is the serine/threonine protein kinase Tor1 in the yeast *Saccharomyces cerevisiae* or mTor in animal cells (Broach 2012; Conrad *et al.* 2014).

Recent advances in our understanding of how TorC1 kinase activity is regulated in yeast derive from studies demonstrating the active participation and requirement of five proteins for TorC1 activation in response to increased concentrations of leucine: Vam6 and two protein complexes consisting of Gtr1-Gtr2 and Ego1-Ego3, respectively (Binda *et al.* 2009; Bonfils *et al.* 2012; Zhang *et al.* 2012; Panchaud *et al.* 2013). Vam6 is a guanine nucleotide exchange factor, or GEF, that mediates conversion of the GDP (guanosine-5′-diphosphate) form of Gtr1 to its active GTP (guanosine-5′-triphosphate) form. Gtr1-GTP-Gtr2 associates with the Ego1-Ego3 complex, which in a yet-to-be-elucidated manner activates TorC1. In these studies, the reporter of TorC1 kinase activation was the phosphorylation of the Sch9 kinase, which is a known regulator of protein synthesis initiation through its phosphorylation of six residues in the C-terminal portion of the protein (Binda *et al.* 2009; Bonfils *et al.* 2012; Zhang *et al.* 2012; Panchaud *et al.* 2013).

Gln3 and Gat1, the nitrogen-responsive, GATA-family transcription activators, also have been extensively used as reporters of TorC1 activity. In nitrogen-replete conditions, Gln3 is cytoplasmic and transcription of genes encoding the proteins required to transport and catabolize poorly used nitrogen sources, *e.g.*, proline, urea or allantoin, is minimal (Cooper 1982, 2004; Hofman-Bang 1999; Magasanik and Kaiser 2002; Broach 2012; Conrad *et al.* 2014). When the supply of readily used nitrogen sources (*e.g.*, yeast extract, peptone, dextrose; glutamine; or ammonia) become exhausted or unavailable, Gln3 relocates to the nucleus, where Gln3-activated transcription increases dramatically so that cells are able to scavenge a broader range of nitrogenous compounds from their environments. This nitrogen-responsive regulation has long been referred to as nitrogen catabolite repression (NCR) (Cooper 1982).

The findings that the transcriptome profiles of NCR-sensitive transcription were significantly similar to those observed after treating cells with the specific TorC1 inhibitor, rapamycin, led to the conclusion that nitrogen-responsive regulation of Gln3 and Gat1 was achieved via TorC1 (Beck and Hall 1999; Cardenas *et al.* 1999; Hardwick *et al.* 1999; Bertram *et al.* 2000; Kulkarni *et al.* 2001; Puria *et al.* 2008). Strengthening this connection was the observation that, in some genetic backgrounds, nuclear Gln3 localization in proline-grown cells or in response to rapamycin treatment required the TorC1 pathway phosphatase Sit4 (Beck and Hall 1999).

However, increasing evidence has demonstrated that TorC1 is unlikely to be solely responsible for the nitrogen-responsive regulation of Gln3 localization and NCR-sensitive transcription (Cox *et al.* 2004a,b; Tate *et al.* 2005, 2006, 2009; Georis *et al.* 2011; Broach 2012; Feller *et al.* 2013; Tate and Cooper 2013; Fayyadkazan *et al.* 2014; Rai *et al.* 2013, 2014). The most recent evidence in support of this idea are the findings that: (i) Gln3 localization is not responsive to intracellular leucine concentrations that modulate TorC1 activity (Tate and Cooper 2013), and (ii) amino acid substitutions in Ure2, the negative regulator of Gln3 that forms a Gln3-Ure2 complex in nitrogen-rich medium and in Gln3 itself, are able to abrogate its response to rapamycin treatment while leaving nitrogen source-dependent Gln3 regulation intact (Feller *et al.* 2013; Rai *et al.* 2013, 2014).

Given this background, it seemed imperative to query directly whether the Gtr1, Gtr2, Ego1/Mse2, and Ego3/Mse1 complex proteins required for TorC1 activation also were required to sequester Gln3 in the cytoplasm of cells cultured in nitrogen-replete conditions. Answering this question is particularly important because TorC1 activation and Gln3 phosphorylation have for 16 years have generally been accepted to be responsible for cytoplasmic Gln3 sequestration and repressed NCR-sensitive transcription (Beck and Hall 1999; Cardenas *et al.* 1999; Puria *et al.* 2008; Hardwick *et al.* 1999; Bertram *et al.* 2000; Magasanik and Kaiser 2002; Broach 2012; Conrad *et al.* 2014). Therefore, one would expect that the proteins required for TorC1 activation would also be required for cytoplasmic Gln3 sequestration and repressed transcription. To address this question, we constructed deletions in the cognate genes for each of the Gtr-Ego complex proteins and then tested Gln3 localization and NCR-sensitive gene expression in these deleted strains. The evidence obtained was remarkable: none of these proteins were required for the long-term sequestration of Gln3-Myc^13^ in the cytoplasm of steady state glutamine-growing cultures or short-term after resupplying excess nitrogen to nitrogen-limited or nitrogen-starved cells. The same results were obtained when NCR-sensitive *GDH2* and *DAL5* transcription were measured: the expression of these genes was minimal in all of the mutants grown in nitrogen-replete conditions. 

These data clearly demonstrated that the response of Gln3 to excess nitrogen was mechanistically independent of Gtr1/2-Ego1/3-dependent TorC1 activation, thus demonstrating the existence of either (i) another mechanism to achieve cytoplasmic Gln3 sequestration in excess nitrogen or (ii) nitrogen-responsive TorC1 activation that does not require the Gtr-Ego complexes. On the other hand, nuclear Gln3 localization in response to nitrogen limitation or starvation was affected adversely to varying degrees in all but the *gtr1*Δ, indicating that these proteins play a newly identified role in the regulation of Gln3 localization other than their roles in TorC1 activation. Together, the data presented give a more complete and detailed view of the regulatory pathways controlling Gln3 localization and function. In addition, they provide solid motivation to search for additional mechanisms of activating TorC1 or TorC1-independent mechanisms of regulating Gln3 localization and function in response to changes in the nitrogen environment.

## Materials and Methods

### Yeast strains and culture conditions

The *S. cerevisiae* strains used in this work appear in Table 1. All deletion mutant strains were derived from TB123. Growth conditions were identical to those described in Tate *et al.* (2009, 2010). Cultures were grown to mid-log phase (A_600 nm_ = 0.5) in YNB (without amino acids or ammonia) minimal medium containing the indicated nitrogen source (final concentration 0.1%). Appropriate supplements (120 μg/mL leucine, 20 μg/mL histidine, 20 μg/mL tryptophan, and 20 μg/mL uracil) were added to cover auxotrophic requirements.

**Table 1:** *Saccharomyces cerevisiae* strains used in this work

Strain	Pertinent Genotype	Complete Genotype
TB123	Wild type Gln3-Myc^13^	*MATa*, *leu2-3*, *112*, *ura3-52*, *trp1*, *his4*, *rme1*, *HMLa*, *GLN3-MYC^13^[KanMX]*
FV359	*gtr2*Δ-Gln3-Myc^13^	*MATa*, *leu2-3*, *112*, *ura3-52*, *trp1*, *his4*, *rme1*, *GLN3-MYC^13^[KanMX] HMLa*, *gtr2*Δ::*natMX*
FV406	*gtr1*Δ-Gln3-Myc^13^	*MATa*, *leu2-3*, *112*, *ura3-52*, *trp1*, *his4*, *rme1*, *GLN3-MYC^13^[KanMX] HMLa*, *gtr1*Δ::*natMX*
FV515	*ego1*Δ-Gln3-Myc^13^	*MATa*, *leu2-3*, *112*, *ura3-52*, *trp1*, *his4*, *rme1*, *GLN3-MYC^13^[HIS3] HMLa*, *ego1*Δ::*natMX*
RR216	*ego3*Δ-Gln3-Myc^13^	*MATa*, *leu2-3*, *112*, *ura3-52*, *trp1*, *his4*, *rme1*, *GLN3-MYC^13^[KanMX] HMLa*, *ego3*Δ::*natMX*

Cells in mid-exponential phase to be transferred from one medium to another were harvested by rapid filtration. The harvested cells were then suspended in fresh, prewarmed and preaerated medium that was the same as the one from which the cells were harvested. When nitrogen (glutamine) was added to a nitrogen-starved culture or a culture that had been previously shifted from glutamine to proline medium, it was added as a solid which quickly dissolved. Between 45 and 60 sec elapsed between the onset of glutamine addition and sampling of the re-fed culture.

### Strain construction

Deletion strains involving insertion of kanMX or natMX cassettes were constructed using the long flanking homology strategy of Wach (1996) using L1−L4 primers listed in Table 2.

**Table 2:** Primers used in strain constructions

Oligonucleotide Designator	Oligonucleotide Sequence
EGO1-L1	5′-GTTTGCAGCGGAACTGTGAA-3′
EGO1-L2	5′-GGGGATCCGTCGACCTGCAGCTTTTACGACTTAAATCTGTCG-3′
EGO1-L3	5′-AACGAGCTCGAATTCATCGATGAACTTTTTGTATAACATCATTGG-3′
EGO1-L4	5′-AGGATGTTTTCCCGGCAAGT-3′
EGO3-L1	5′-GGCAGTTATCAGCAGCAAACGGTATCCAAAATATTGAAGCAATATGCCTTGACAGTCTTGACGTGC-3′
EGO3-L2	5′-GAGACGCATGAAAAGGTGTGGCCTCGATAAATATTGTTATTCCCATCACGCACTTAACTTCGCATCTG-3′
GTR1-L1	5′-TCTCCCCTTCCGGTTGTGTC-3′
GTR1-L2	5′-GGGGATCCGTCGACCTGCAGCCATTACTAAATTGTCGATTGATAAACGTGATTTTG-3′
GTR1-L3	5′-CGAGCTCGAATTCATCGATGATGACTGAGGTGAGTAGACGAAACATTCGGCAATTG-3′
GTR1-L4	5′-CCGGTGGTGGCTTAATGACC-3′
GTR2-L1	5′-TTTACCATTGTTATATTTTCTCG-3′
GTR2-L2	5′-GGGGATCCGTCGACCTGCAGCCATGTTGTATGTGTATTAGTACCGTTGTCCTGGAG-3′
GTR2-L3	5′-CGAGCTCGAATTCATCGATGATGAAAGACGTAAGGCATGAAAATATTAGGG-3′
GTR2-L4	5′-GCTGTTGTACCGTCCGTACG-3′

### Indirect immunofluorescence microscopy

Cell collection and immunofluorescent staining were performed as previously described (Tate *et al.* 2006, 2009; Georis *et al.* 2008; Feller *et al.* 2013). Stained cells were imaged using a Zeiss Axioplan 2 imaging microscope with a 100× Plan-Apochromat 1.40 oil objective at room temperature. Images were acquired using a Zeiss Axio camera and AxioVision 3.0 and 4.8.1 (Zeiss) software. For presentation, images were processed with Adobe Photoshop and Illustrator programs. Level settings (shadow and highlight only) were altered where necessary to avoid any change or loss in cellular detail relative to what was observed in the microscope; changes were applied uniformly to the image presented and were similar from one image to another.

### Determination of intracellular Gln3-Myc^13^ distribution

We quantitated intracellular Gln3-Myc^13^ localization by manually scoring its localization in 200 or more cells in multiple, randomly chosen fields from which each image presented was taken. Scoring was performed exclusively using unaltered, primary .zvi image files viewed with Zeiss AxioVision 3.0 and 4.8.1 software.

Cells were classified into one of three categories: cytoplasmic (cytoplasmic Gln3-Myc^13^ fluorescence only; red bars in the histograms), nuclear-cytoplasmic (Gln3-Myc^13^ fluorescence appearing in the cytoplasm as well as co-localizing with DAPI-positive material; yellow bars), and nuclear (Gln3-Myc^13^ fluorescence co-localizing only with DAPI-positive material; green bars). Representative “standard” images demonstrating the differences in these categories are shown in Figure 2 of Tate *et al.* 2009, along with a description of how the scoring criteria were applied. Time-course experiments of the kind presented here do not easily lend themselves to statistical analysis because even small experiment-to-experiment shifts in the overall shape of the response curves destroy the apparent precision of the individual measurements. However, experiments were repeated two or more times with similar results. The reproducibility of our scoring has been evaluated repeatedly (Tate *et al.* 2006, 2010; Rai *et al.* 2014). In the latest evaluation, we analyzed the data for three different experimental conditions, each from 10 different experiments performed over a nine month period. The maximum standard deviation observed in our scoring was ± ∼7–8% (Rai *et al.* 2014). Images accompanying the histograms were selected on the basis that they exhibited intracellular Gln3-Myc^13^ distributions as close as possible to those observed in the quantitative scoring data.

### Quantitative reverse-transcription real time polymerase chain reaction analyses

RNA isolation and cDNA synthesis were conducted as described by Georis *et al.* (2009). Specific DNA targets were quantified by RT polymerase chain reaction performed on a StepOnePlus device (Applied Biosystems, Foster City, CA) using *DAL5*, *GDH2*, and *TBP1* primers described previously (Georis *et al.* 2008, 2011). The values reported represent the averages of at least two experiments from independent cultures; error bars indicate SEs.

## Results

Our previous work demonstrating that the model describing nitrogen-responsive regulation of TorC1 activity was insufficient to account for control of the GATA factors Gln3 and Gat1 prompted an important question that directly tests whether or not nitrogen-dependent TorC1 activation, as we currently understand it, regulates GATA factor localization and function. Do the Gtr1/Gtr2 and Ego1/Ego3 complexes, required for TorC1 activation and downstream Sch9 phosphorylation in nitrogen-replete medium, also participate in cytoplasmic Gln3 sequestration during nitrogen excess and/or nuclear Gln3 localization and subsequently Gln3-dependent gene expression during nitrogen limitation/starvation? To frame these questions in a context that permitted overall comparisons of present Gln3 localization data with that reported for growth recovery and Sch9 phosphorylation (Binda *et al.* 2009), we used an experimental format similar to the one originally employed to establish that Gtr1, Gtr2, Ego1, and Ego3 were required for recovery from rapamycin-induced growth arrest and TorC1 activation. In those investigations, wild-type and mutant cells, growing in complex nitrogen-rich medium, were treated with rapamycin for 6 hr to mimic nutrient starvation. The cells were then transferred to rapamycin-free rich (YPD, yeast extract, peptone, dextrose) medium where growth recovery was measured (Figure 1 of Binda *et al.* 2009). Sch9 phosphorylation also was measured in nitrogen-replete medium. Our experiments differed from those of Binda *et al.* (2009) in that we used a defined, nitrogen-rich medium (YNB-glutamine) and naturally occurring conditions, *i.e*., short and long-term nitrogen starvation and transfer from nitrogen-rich to nitrogen-poor media or vice-versa rather than rapamycin treatment.

**Figure 1:**
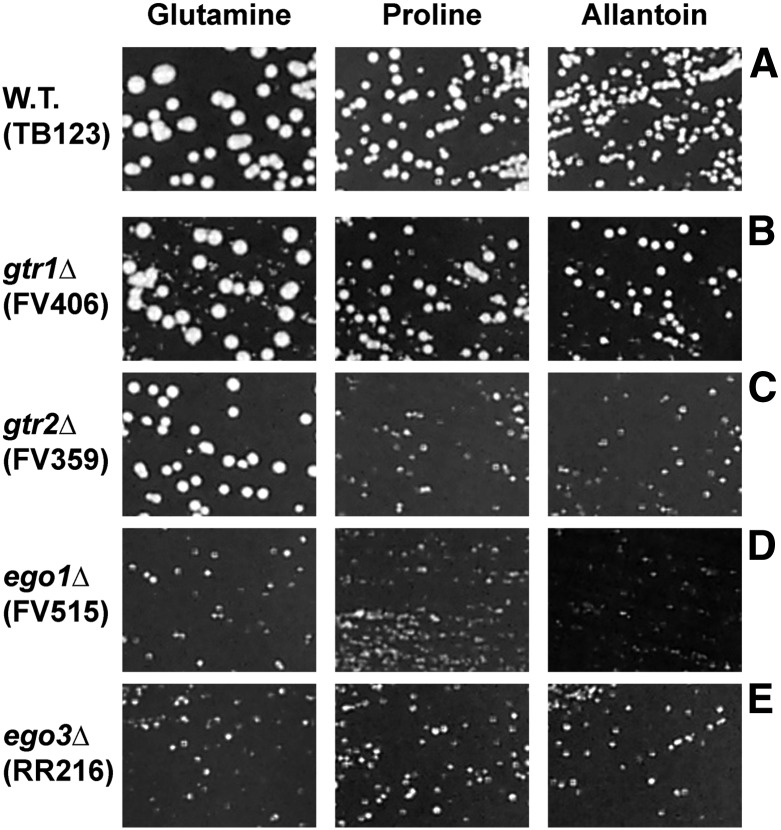
Requirements of Gtr1 (B), Gtr2 (C), Ego1 (D), and Ego3 (E) for growth with repressive (glutamine) and derepressive (proline and allantoin) nitrogen sources. Wild-type (A) and mutant cells were streaked on the same plates containing YNB-glutamine, -proline or -allantoin medium. Cultures were incubated at 30° for 72 hr and photographed. Strain numbers and pertinent genotypes are indicated to the left of the images.

To address the aforementioned questions, we deleted each of the four genes (*GTR1*, *GTR2*, *EGO1*, *EGO3*), verified that the production of the cognate mRNAs for each of them was abolished in the mutant cells (data not shown) and assayed their effects on growth in the presence of rich (glutamine) and poor (proline and allantoin) nitrogen sources (Figure 1). Three of the four mutant strains exhibited poor growth when proline or allantoin was provided as sole nitrogen source (Figure 1, A, C, D, and E). Growth of the *gtr1*Δ, however, did not slow to nearly the degree observed for the other three mutants with these nitrogen sources, but there was heterogeneity in the *gtr1*Δ colony sizes. Further, even with glutamine as sole nitrogen source, the growth of the *ego1*Δ and *ego3*Δ mutants was diminished relative to wild type (Figure 1, A, D, and E). In contrast, the growth of *gtr2Δ* mutant was only affected in the presence of poor nitrogen sources (Figure 1C).

### Kinetics of nuclear Gln3-Myc^13^ localization after the onset of nitrogen starvation or limitation

**Figure 2:**
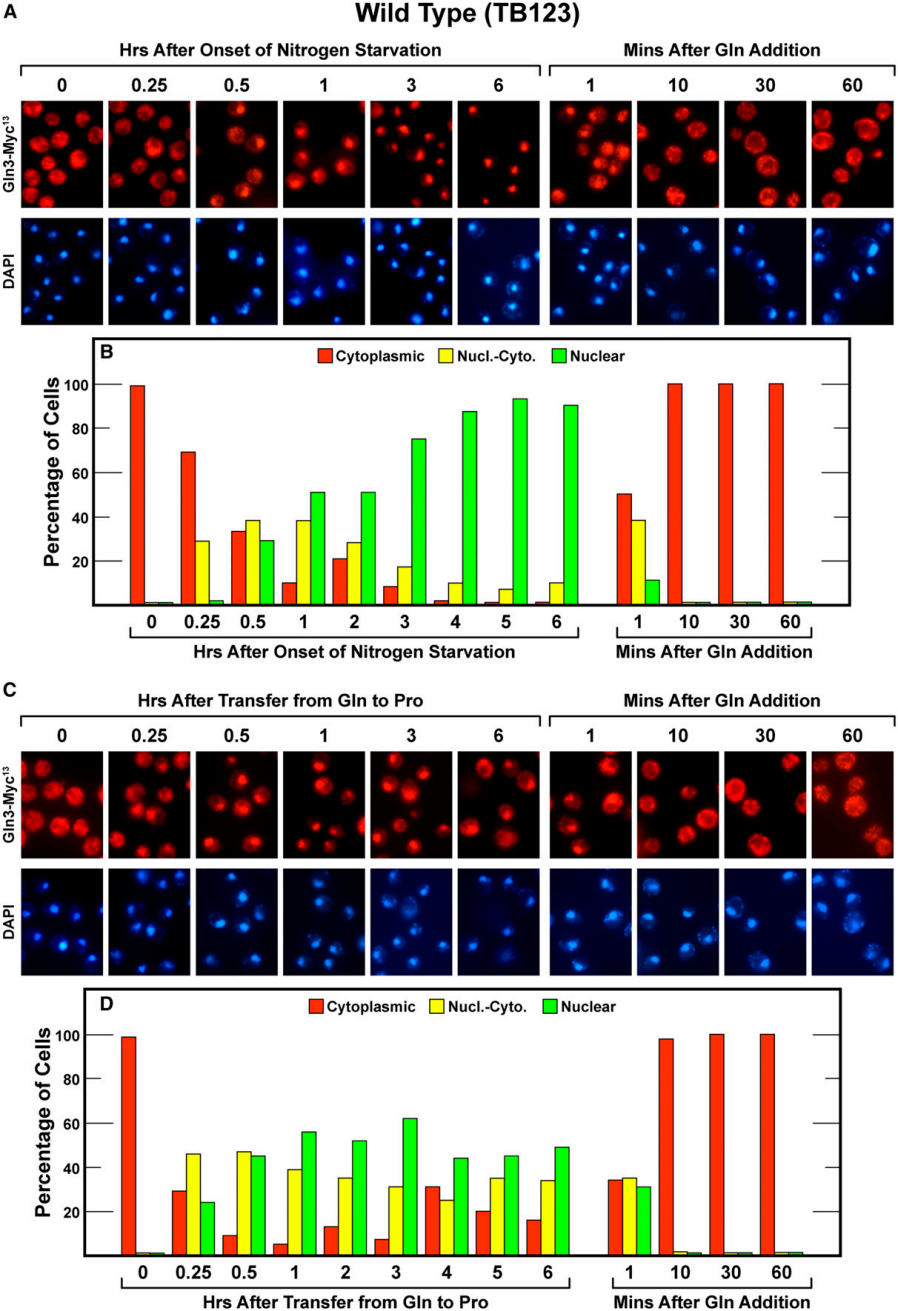
Time course of Gln3-Myc^13^ localization in wild-type cells (TB123) after nitrogen starvation (A and B) or transfer from nitrogen-replete to nitrogen-limiting medium (C and D), and resupplying excess nitrogen to all of the cultures (A−D). Cultures were originally grown to mid-log phase (A_600 nm_ = 0.5) in YNB-glutamine medium. At zero time, the first culture was sampled and the remainder transferred to nitrogen-free-YNB medium (A and B) and the second culture was sampled at zero time and the remainder transferred to YNB-proline medium (C and D). Samples of each culture were taken as indicated. After 6 hr of incubation, glutamine was added to each culture (final concentration of 0.1%) and sampling continued for an additional hour. Samples were then processed for indirect immuno-fluorescence microscopy as described in the section *Materials and Methods*. Time-course experiments as presented here do not easily lend themselves to statistical analysis because even small experiment to experiment shifts in the overall shape of the response curves destroy the precision of the individual measurements. However, we have repeatedly assessed the precision of our scoring; see the section *Methods and Materials* and Tate *et al.* (2006, 2010) and Rai *et al.* (2014).

When wild-type (TB123) cells containing Gln3-Myc^13^ were transferred from YNB-glutamine to YNB-nitrogen free medium, Gln3-Myc^13^ began relocating from the cytoplasm to the nucleus within 15 min and became largely nuclear within 3 hr (Figure 2, A and B, left side). When these cells were resupplied with excess nitrogen, by adding glutamine to the nitrogen-free medium, Gln3-Myc^13^ immediately relocated from the nucleus to the cytoplasm. In fact, Gln3-Myc^13^ relocation to the cytoplasm was so rapid that it was already half complete before we could collect our first sample (∼1 min.). It totally relocated within 10 min (Figure 2, A and B, right side), and then remained completely cytoplasmic over the next hour.

Transferring cells from nitrogen-replete to nitrogen-limiting conditions, *i.e.*, from glutamine to proline medium, generated a less strong response. Gln3-Myc^13^’s initial entry into the nucleus occurred more quickly than with nitrogen starvation in that maximum relocation was largely achieved within 30 min (Figure 2, C and D, left side). The degree of nuclear localization was similar to that observed in short-term nitrogen starvation (1−2 hr). However, the overall extent of Gln3-Myc^13^ relocation was less than seen with nitrogen starvation. In contrast, the response to refeeding glutamine to cells growing in proline medium was exactly the same as occurred when refeeding nitrogen-starved cells. Gln3-Myc^13^ began relocating to the cytoplasm immediately and was completely cytoplasmic within 10 min (Figure 2, C and D, right side).

### Effects of deleting GTR1 and GTR2 on Gln3-Myc^13^ movement in and out of the nucleus

**Figure 3:**
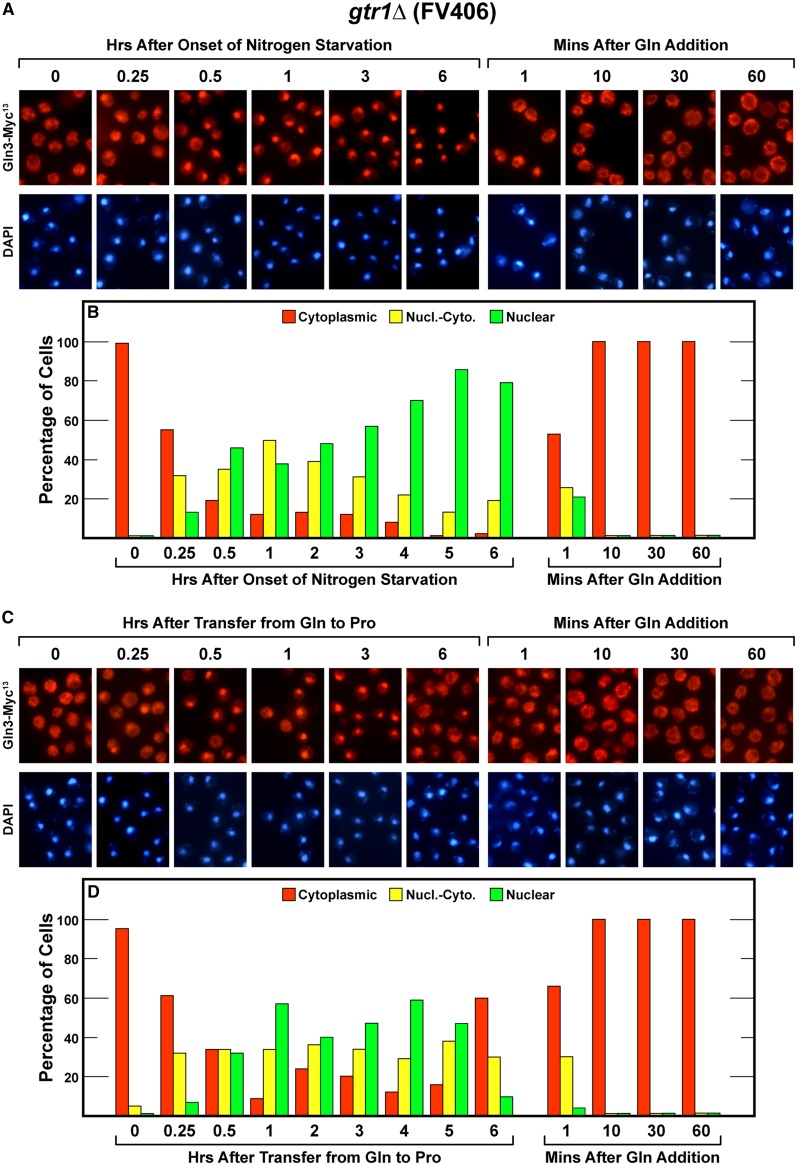
Time course of Gln3-Myc^13^ localization in *gtr1*Δ (FV406) cells after nitrogen starvation (A and B), transfer from nitrogen-replete to nitrogen-limiting medium (C and D), and resupplying excess nitrogen to all of the cultures (A−D). The experimental format and treatment of the samples were identical to those in Figure 2.

The first mutant analyzed was a *gtr1*Δ (FV406). Consistent with the growth data (Figure 1), there was no demonstrable effect of deleting *GTR1* other than a small shift in the kinetics of Gln3-Myc^13^ movement from the cytoplasm to the nucleus during short-term nitrogen limitation or long-term nitrogen starvation (Figure 3, A and B). Otherwise, the *gtr1*Δ exhibited a wild-type phenotype (Figure 2 *vs.* Figure 3, A and B). We also were unable to demonstrate a significant change in the behavior of Gln3-Myc^13^ localization for up to 5 hr after transferring *gtr1*Δ cells from a rich to a poor nitrogen source (Figure 3, C and D). Some Gln3-Myc^13^ movement out of the nucleus did occur, however, between the 5th and 6th hours after the transfer to proline medium; the significance of this observation is unclear.

On the other hand, addition of glutamine to nitrogen-starved or nitrogen-limited *gtr1*Δ cells elicited rapid and dramatic exit of Gln3-Myc^13^ from the nucleus. Gln3-Myc^13^ was cytoplasmic or nuclear-cytoplasmic in more than half of the cells before the first sample could be collected irrespective of whether glutamine was added to nitrogen-starved *gtr1*Δ cells or in those provided with a derepressive nitrogen source (Figure 3, right side). Thus, Gtr1 was not required for cytoplasmic Gln3-Myc^13^ sequestration in glutamine re-fed cells. Similarly, Gtr1 was not required for cytoplasmic Gln3 sequestration when the cells were growing logarithmically in nitrogen-rich glutamine medium, a growth condition where TorC1 is activated (zero time points in Figure 3).

**Figure 4:**
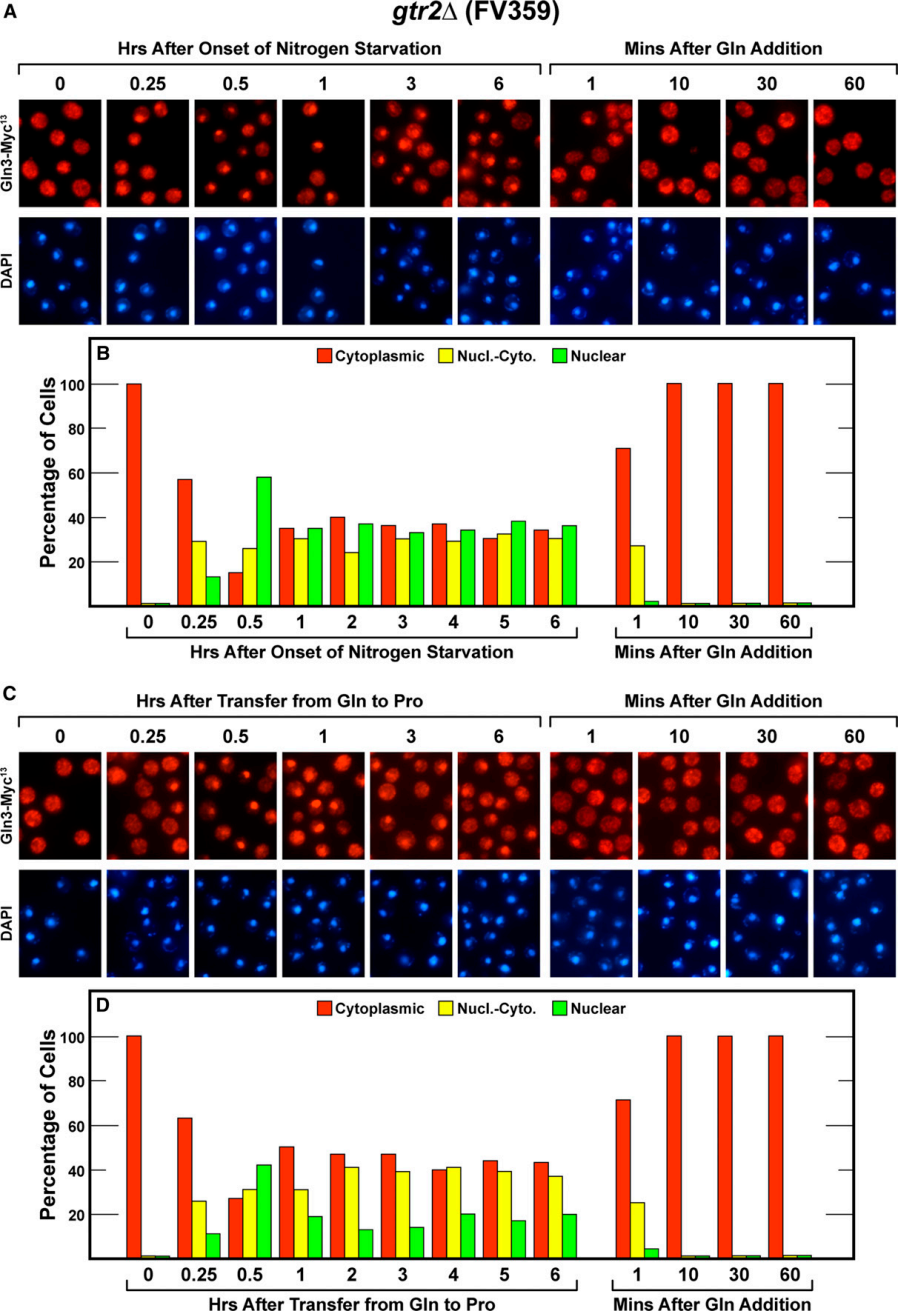
Time course of Gln3-Myc^13^ localization in *gtr2*Δ (FV359) cells after nitrogen starvation (A and B), or transfer from nitrogen-replete to nitrogen-limiting medium (C and D), and resupplying excess nitrogen to all of the cultures (A−D).

Gtr1 and Gtr2 are members of a heterodimeric complex that associates with the Ego1/Ego3 complex also reported to be required for TorC1 activation (Binda *et al.* 2009; Bonfils *et al.* 2012; Zhang *et al.* 2012; Panchaud *et al.* 2013). As such, we expected *gtr1* and *gtr2* deletions to exhibit similar phenotypes. Therefore, we were somewhat surprised to find that their phenotypes were different. Short- and long-term nitrogen-starved and nitrogen-limited (transferred to proline for 6 hr) *gtr2*Δ (FV359) cells behaved similarly (Figure 4, A−D). In all three cases, alteration of the cells’ nitrogen supplies elicited rapid initial Gln3-Myc^13^ movement into the nucleus; the effects were clearly evident within 30 min of altering the medium (Figure 4). However, further Gln3-Myc^13^ relocation then abruptly ceased and remained at this intracellular distribution for the duration of the experiment with Gln3-Myc^13^ situated equally in the nuclear, nuclear-cytoplasmic and cytoplasmic scoring categories (Figure 4).

There were two conditions where the phenotypes of the *gtr1*Δ and *gtr2*Δ mutants exhibited striking and identical phenotypes. Cytoplasmic Gln3-Myc^13^ sequestration after the addition of glutamine to nitrogen-limited and -starved cultures did not require functional Gtr1 and Gtr2 (Figure 3 and Figure 4, right side). The same absence of a Gtr2 requirement was also observed in logarithmically, glutamine-growing cells (Figure 4, left side, zero time points).

### Effects of deleting EGO1 and EGO3 on Gln3-Myc^13^ localization

**Figure 5:**
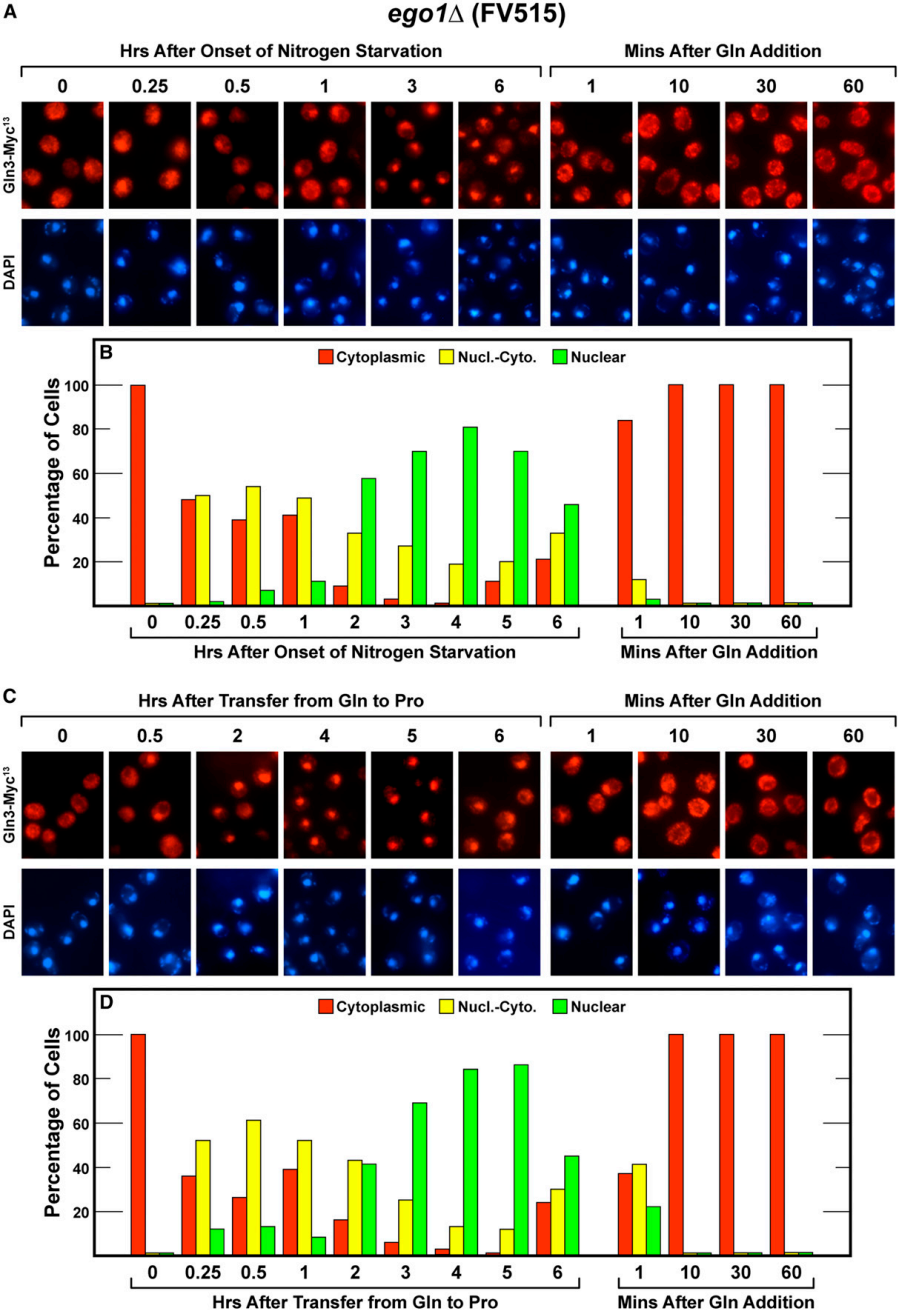
Time course of Gln3-Myc^13^ localization in *ego1*Δ (FV515) cells after nitrogen starvation (A and B), or transfer from nitrogen-replete to nitrogen-limiting medium (C and D), and resupplying excess nitrogen to all of the cultures (A−D).

The next complex in the cascade required for TorC1 activation, consists of Ego1 and Ego3 (Binda *et al.* 2009; Bonfils *et al.* 2012; Zhang *et al.* 2012; Panchaud *et al.* 2013). There were several measurable effects of losing Ego1 on cells starved for nitrogen or transferred from excess to growth limiting proline medium. First, nuclear Gln3-Myc^13^ localization in response to short-term nitrogen starvation was lower than in wild type (compare Figures 2, A and B with Figure 5, A and B, 0−1 hr). Although the Gln3-Myc^13^ response to long-term nitrogen starvation was initially the same as in wild type cells, nuclear Gln3-Myc^13^ localization was not sustained as nitrogen starvation extended beyond 4 hr (Figure 5, A and B). Gln3-Myc^13^ began exiting the nucleus between the fifth and sixth hours. However, the significance of this observation is uncertain.

The initial response of shifting *ego1*Δ cells from glutamine to proline medium was much the same as occurred with short-term nitrogen starvation, again a smaller response than observed in wild type during the first hr. Thereafter, up to 5 hr, Gln3-Myc^13^ became more nuclear than occurred in wild type as though the cells were becoming increasingly starved even though they were being cultured in a stable condition with proline as sole nitrogen source. Then, as with long-term starvation, Gln3-Myc^13^ began exiting from the nucleus between hrs five and six (Figure 5, C and D).

As occurred with the previous two deletion mutants, we could demonstrate no Ego1 requirement for relocating Gln3-Myc^13^ from the nucleus to the cytoplasm when glutamine was added to re-feed the nitrogen-starved and nitrogen-limited cultures (Figure 5, A−D, right side). Supporting this result, we observed no requirement of Ego1 to sequester Gln3 in the cytoplasm of cells growing logarithmically in YNB-glutamine medium (Figure 5, A−D, left side, zero time point).

**Figure 6:**
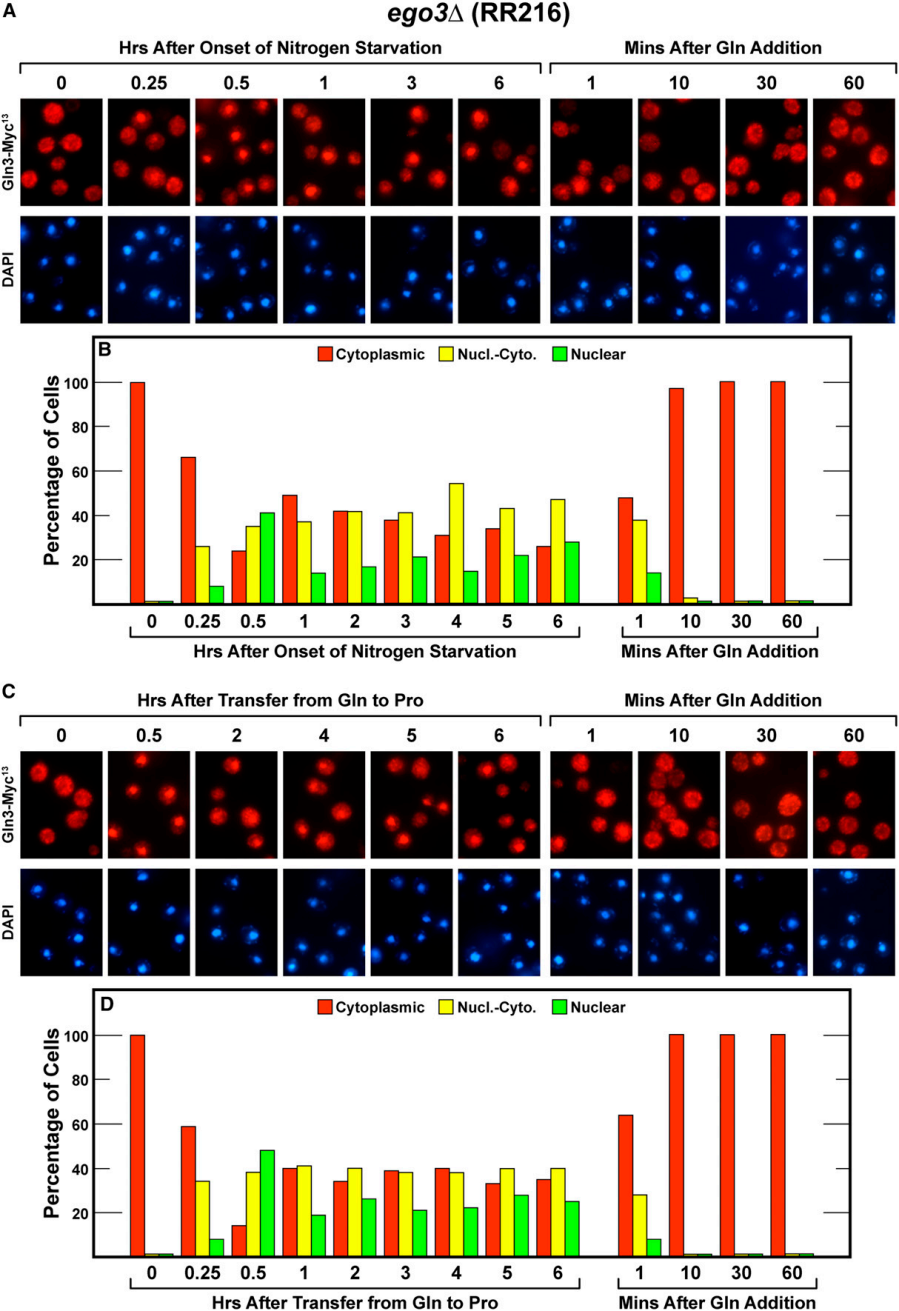
Time course of Gln3-Myc^13^ localization in *ego3*Δ (RR216) cells after nitrogen starvation (A and B), or transfer from nitrogen-replete to nitrogen-limiting medium (C and D), and resupplying excess nitrogen to all of the cultures (A−D).

The final TorC1 activation pathway component we investigated was Ego3. For the first half hour of nitrogen starvation, the response of Gln3-Myc^13^ in an *ego* was largely wild type, Gln3-Myc^13^ began relocating from the cytoplasm to the nucleus (Figure 6, A and B, left side). At that point, however, Gln3-Myc^13^ movement ceased with it remaining more or less equally distributed in the three localization scoring categories just as occurred when the *gtr2*Δ was nitrogen starved (compare Figure 6, A and B with Figure 4, A and B). Long-term nitrogen starvation-elicited nuclear Gln3-Myc^13^ localization did not occur. Again, Gln3-Myc^13^ was distributed equally in the three scoring categories. When the *ego3*Δ was transferred from glutamine to proline medium, the phenotype was the same as with nitrogen starvation and as previously observed with the *gtr2*Δ.

At this point, we were no longer surprised by our inability to demonstrate a requirement of Ego3 for Gln3-Myc^13^ to be relocated from the nucleus to the cytoplasm after re-feeding of glutamine to nitrogen-starved cultures or a culture growing in proline medium. And as before, Ego3 was not required to sequester Gln3-Myc^13^ in the cytoplasm of steady state glutamine-grown cells (Figure 6, zero time points). In summary, none of the TorC1 activation pathway components previously shown to be required for TorC1-dependent Sch9 phosphorylation were required for cytoplasmic Gln3 sequestration in steady state glutamine-grown cultures, or when glutamine was added to nitrogen-starved cultures or those provided with a poor nitrogen source.

### Effects of deleting Gtr-Ego complex components on Gln3-Myc^13^ localization in steady-state cultures provided with proline as the sole nitrogen source

**Figure 7:**
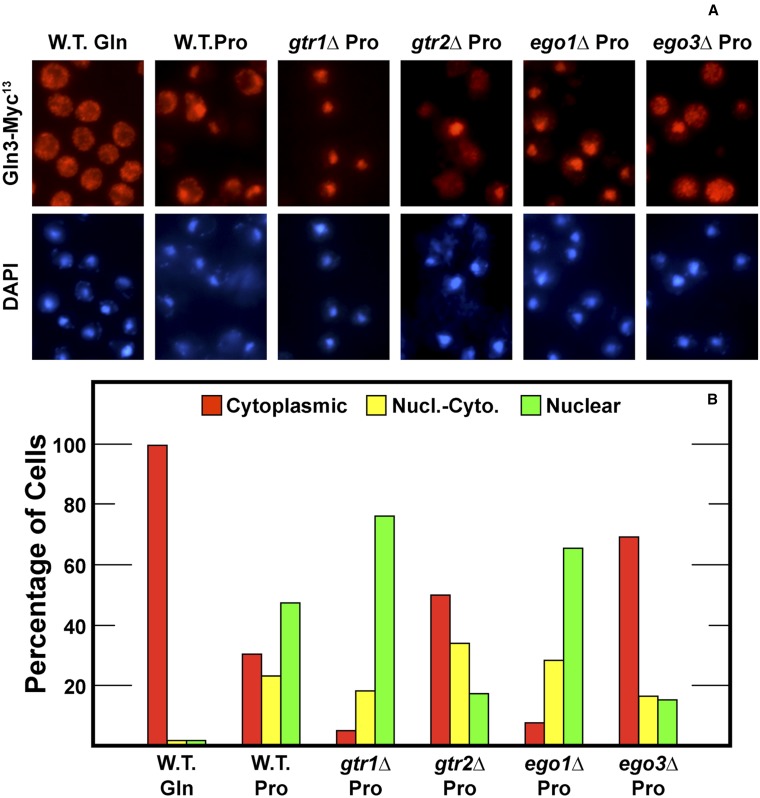
Gtr2 and Ego3 are required for nuclear Gln3-Myc^13^ localization for steady state growth in minimal-proline medium. Wild-type (TB123), *gtr1*Δ (FV406), *gtr2*Δ (FV359), *ego1*Δ (FV515), and *ego3*Δ (RR216) were grown of a density of A_600 nm_ = 0.5-0.55 in YNB-proline medium. Intracellular distribution of Gln3-Myc^13^ was then determined in each of the cultures as described in the section *Materials and Methods*.

Aforementioned experiments demonstrated Gtr2, Ego1, and Ego3 were required to varying degrees for nuclear Gln3-Myc^13^ localization when cells were shifted from glutamine to proline medium. It was conceivable, however, these requirements resulted from insufficient time being allowed for the transition from nitrogen-replete to nitrogen-poor medium and would disappear over time. To assess this possibility, wild-type and mutant cultures were grown in proline medium (Figure 7). Both Gtr2 and Ego3 were required for high-level nuclear Gln3-Myc^13^ localization, supporting conclusions reached during the time course studies. When the steady-state Gln3 localization profiles were compared with the values observed at 4−6 hr, data for *ego1*Δ correlated relatively well, but there was an even greater Ego3 requirement in proline-grown cells than at 4−6 hr after they were transferred from glutamine to proline medium (compare Figure 5, Figure 6, and Figure 7). As observed in cells transferred to proline, *GTR1* was not required for Gln3 localization in proline-grown cells.

The Gln3-Myc^13^ localization profiles in wild type, *gtr1*Δ, and *gtr2*Δ strains paralleled the growth of these strains in proline medium (compare Figure 1 and Figure 7). There was, however, less positive correlation when *ego1*Δ and *ego3*Δ mutants were analyzed. Gln3-Myc^13^ localization in steady-state proline-grown cells would lead to the prediction that the *ego1*Δ would grow well, whereas the *ego3*Δ would not (compare Figure 7 with Figure 1). In fact, both *ego1*Δ and *ego3*Δ mutants grew poorly in proline (Figure 1). Importantly, both mutants also grew poorly in glutamine medium where Gln3-Myc^13^ is cytoplasmic and NCR-sensitive transcription is minimal. This finding strongly suggests that the growth of these mutants was dependent on more than Gln3-Myc^13^ localization, which likely accounts for the poorer correlation between Gln3-Myc^13^ localization and growth of the *ego1*Δ and *ego3*Δ mutants.

### Repressed reporter gene expression is unaffected in Gtr-Ego complex mutants

**Figure 8:**
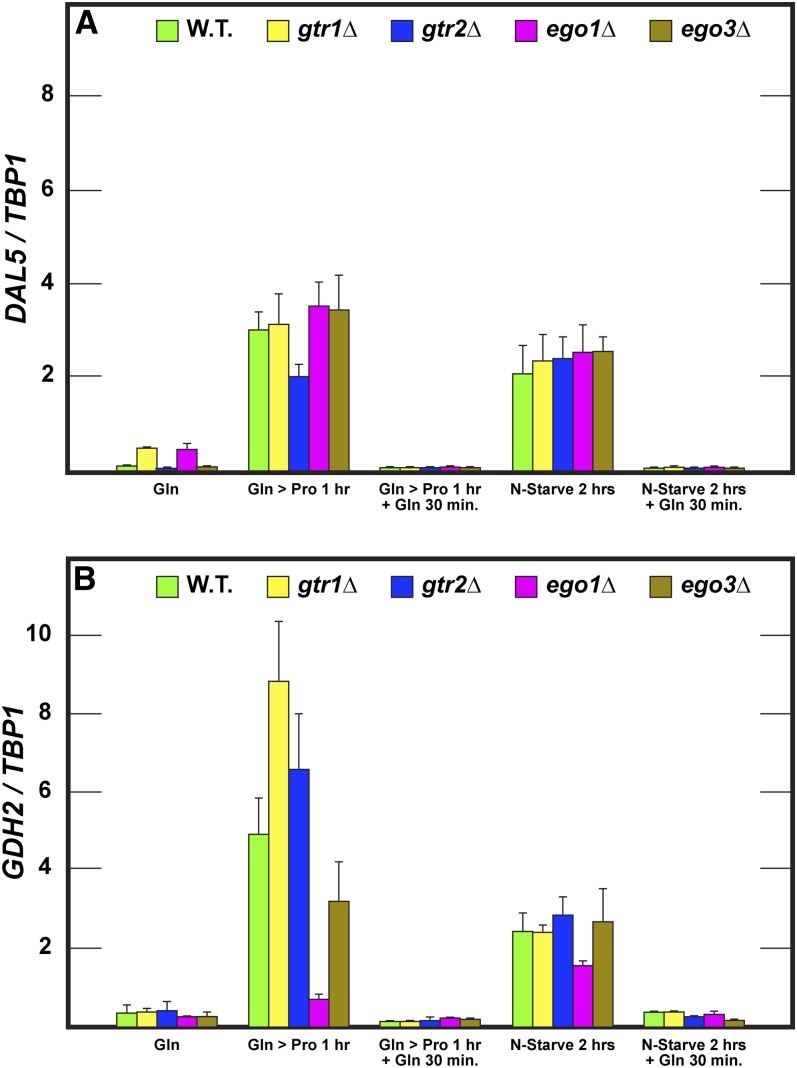
Transcriptional responses to nitrogen excess, limitation and starvation are unaffected in the Gtr/Ego complex mutants. Total RNA was isolated from *GLN3-MYC*^13^ wild-type (TB123), *gtr1*Δ (FV406), *gtr2*Δ (FV359), *ego1*Δ (FV515), and *ego3*Δ (RR216) cells grown in YNB-glutamine medium to mid-exponential phase, transferred to nitrogen-free medium or to proline-containing medium, with or without glutamine re-feeding. Incubation times are indicated on the figure. *DAL5* (A) and *GDH2* (B) mRNA levels were quantified as described in the section *Materials and Methods*

We and others often use reporter gene transcription to evaluate nitrogen-responsive regulation in yeast and other organisms. Therefore, we extended the aforementioned studies by determining the extent to which *DAL5* and *GDH2* transcription correlated with the Gln3-Myc^13^ localization responses in the various mutants. After 2 hr of nitrogen starvation, *DAL5* and *GDH2* expression markedly increased relative to that in glutamine-grown cells, positively correlating with increased Gln3-Myc^13^ localization (Figure 8, A and B), irrespective of whether the assays were performed in wild type, *gtr1*Δ, *gtr2*Δ, *ego1Δ*, or *ego3*Δ mutant strains. A similarly positive correlation was observed when wild-type or mutant cells were transferred from glutamine to proline medium (Figure 8, Gln > Pro 1 hr). Thus, deleting components of the Gtr-Ego complex did not demonstrably affect overall NCR-sensitive gene expression. There was, however, one striking lack of correlation between Gln3-Myc^13^ localization and NCR-sensitive gene expression. It was the low *GDH2* level after shifting *ego1*Δ cells from glutamine to proline medium. The reason for this discrepancy isn’t known. We also observed a minor lack of correlation between Gln3-Myc^13^ localization and NCR-sensitive gene expression. It occurred with *DAL5* expression in the *gtr1*Δ and *ego1*Δ mutants during steady-state growth in glutamine medium. Transcription was modestly greater in the mutants than wild type yet Gln3-Myc^13^ localization was similarly sequestered to the cytoplasm in all three cases. It is difficult to know whether this difference in transcription is meaningful given how close it was to basal levels, but in any case the reason for the difference is unknown.

There was an important correlation between Gln3-Myc^13^ localization and *DAL5* and *GDH2* transcription. The two processes responded in absolute lock-step when wild-type and mutant cells were growing in steady state with nitrogen-rich glutamine as the nitrogen source. Gln3-Myc^13^ was totally excluded from the nucleus under these conditions and *DAL5* and *GDH2* transcription was just barely detectable. The same outcome occurred when glutamine was re-fed to nitrogen-starved cells or those growing in a derepressive nitrogen source (Figure 8). Together, these data indicated little if any required participation of Gtr1, Gtr2, Ego1, or Ego3 in the responses of overall *DAL5* and *GDH2* gene expression to nitrogen limitation or starvation. More importantly, they unequivocally demonstrated that these proteins were completely dispensable when it came to short- or long-term control of Gln3-Myc^13^ localization and function in cells provided with excess nitrogen either in steady state or transition.

## Discussion

### Multiple mechanisms participate in the Gln3 response to nitrogen excess

The data presented clearly demonstrate cytoplasmic Gln3-Myc^13^ sequestration in logarithmically growing cells provided with excess nitrogen or upon refeeding excess nitrogen to nitrogen-starved cells or those growing with a poor nitrogen source does not require Gtr1/2-Ego1/3-dependent TorC1 activation. GATA factor-mediated, NCR-sensitive *DAL5* and *GDH2* transcription closely correlate with Gln3 localization, further supporting the conclusion. These strikig observations raise two pivotal questions about TorC1 activation/inactivation and its role in the regulation of Gln3 localization and function. (i) Is there more than one way that TorC1 can be activated, both short and long term, by the cell’s nitrogen supply? (ii) Is Gln3-Myc^13^ localization and function determined by mechanisms beyond nitrogen-responsive TorC1 activation/deactivation, a previously suggested possibility2 (Cox *et al.* 2004a,b; Tate *et al.* 2005, 2006, 2009; Tate and Cooper 2013; Georis *et al.* 2011; Feller *et al.* 2013; Rai *et al.* 2013, 2014)?

Considering the first question, two possibilities are pertinent. Binda *et al.* (2009) observed a very small amount of residual Sch9 phosphorylation (TorC1 activation) when *gtr1*Δ, *gtr2*Δ, *ego1*Δ, *ego3*Δ, and *tco89*Δ mutants were growing in nitrogen-rich medium. Therefore, one could conceivably argue this residual activity was sufficient to trigger cytoplasmic Gln3 sequestration. However, this interpretation depends on two premises: (i) that the observed Sch9 phosphorylation was in fact catalyzed exclusively by TorC1 kinase in the absence of rapamycin and (ii) cytoplasmic Gln3 sequestration is exquisitely sensitive to TorC1 kinase activity. Without a double mutant (Gtr-Ego complex component and unknown component responsible for residual TorC1 activation) abolishing detectable Sch9 phosphorylation, this possibility cannot be rigorously evaluated. We do not particularly favor the second premise because three hours were required after increasing the intracellular pools of amino acids with 200 μg/mL cycloheximide, conditions that abolish protein synthesis elongation and strongly activate TorC1 kinase, for ∼85% of Gln3 to become cytoplasmic (Tate and Cooper 2013). In contrast Sch9 phosphorylation increased dramatically under similar conditions after a 30 min. 25 μg/ml of cycloheximide treatment (Binda *et al.* 2009).

Alternatively and more likely there exists a means of activating TorC1 in response to nitrogen availability that does not involve the Gtr-Ego complexes. Although this possibility might explain some of our observations, it would not explain a significant number of others. For example, if there is a Gtr-Ego complex-independent TorC1 activation pathway regulating Gln3-Myc^13^ localization, it would have to support both short-term TorC1 activation in response to re-feeding excess nitrogen to nitrogen-limited or -starved cells as well as long-term activation in response to steady state growth in nitrogen-replete environments. Further, if there is a Gtr1/2-Ego1/3 complex-independent way of activating TorC1, we would have to conclude that the outcomes of the two routes of TorC1 activation, *i.e.*, phosphorylation of Sch9 and cytoplasmic sequestration of Gln3-Myc^13^ and down regulation of GATA factor-mediated transcription also follow separable routes of implementation because Sch9 phosphorylation requires all of the proteins studied in this work, whereas cytoplasmic Gln3-Myc^13^ sequestration on re-feeding glutamine or in steady state growth in glutamine medium required none of them.

Alternatively, we prefer to suggest that our data likely indicate the existence of a separate means of implementing cytoplasmic sequestration of Gln3 in the presence of excess nitrogen that does not involve TorC1. Several observations support this idea. We have previously shown that mutations in *URE2* and *GLN3* themselves are able to abrogate a response to rapamycin treatment without adversely affecting NCR-sensitive Gln3 regulation (Feller *et al.* 2013; Rai *et al.* 2013). Further, amino acid substitutions that abolish the Gln3-Tor1 interaction do not result in constitutively nuclear Gln3 localization as expected in nitrogen-rich medium where TorC1 is active (Rai *et al.* 2013). Rather, Gln3 becomes only partially nuclear exhibiting a distinct intracellular distribution in which Gln3 is about equally distributed in the three scoring categories used to assess its localization. This is precisely the behavior that would be expected if one of two mechanisms participating in cytoplasmic Gln3 sequestration were inactivated.

### Gtr2 and Ego3 participation in nuclear Gln3 localization in response to nitrogen limitation or starvation

Our results also demonstrate that loss of Gtr2 and Ego3 diminished the responses of Gln3-Myc^13^ localization to nitrogen availability in a way very similar to that observed in *gln3* amino acid substitution mutants in which the Gln3-Tor1 interaction was abolished (Rai *et al.* 2013). This would again be expected if Gtr2 and Ego3 were participating in only one of two or more Gln3 regulatory mechanisms. There is an unexplained issue, however, in these data. Gtr2 and Ego3 are both members of complexes, Gtr1/2 and Ego1/3. Normally one would expect that abolishing one member of a two-member complex would generate a functional phenotype similar to abolishing the other member. This, of course, did not occur with Gln3-Myc^13^ localization. Deleting *GTR1* had no demonstrable effect on Gln3-Myc^13^ localization and although deleting *EGO1* did affect nuclear Gln3-Myc^13^ localization, the response profile was not the same as in the *gtr2*Δ and *ego3*Δ. The above expectation, however, derives from the assumption that the Gtr and Ego proteins can function only as members of their respective complexes. The Gln3-Myc^13^ data raise the possibility that Gtr and Ego proteins may be able to function both in complexes and as individual molecules or members of yet to be discovered additional complexes. From this perspective, it is interesting that deletion of either *GTR2* or *EGO3* yielded similar phenotypes raising the possibility that they are participating in the same overall process leading to nuclear Gln3 localization.

### Gln3-Myc^13^ localization and Gln3 supported transcription in *gtr1*Δ, *gtr2*Δ, *ego1*Δ, and *ego3*Δ mutants

Where it counted most, Gln3-Myc^13^ localization and nitrogen-responsive *DAL5* and *GDH2* reporter gene transcription closely correlated. Indeed, irrespective of the deletion mutant assayed, Gln3-Myc^13^ was sequestered in the cytoplasm of steady state glutamine-grown cells or when glutamine was added to nitrogen limited/starved cultures, and *DAL5* and *GDH2* transcription was minimal.

There is a final important point that should not be overlooked. The landmark investigation of the Gtr1/2-Ego1/3 complexes used Sch9 phosphorylation as the proxy of TorC1 activity (Binda *et al.* 2009; Bonfils *et al.* 2012; Zhang *et al.* 2012; Panchaud *et al.* 2013), Sch9 being a central regulator of protein synthesis (Powers 2007; Urban *et al.* 2007). We, on the other hand, used intracellular Gln3-Myc^13^ localization to measure responses to various nitrogen environments. Gln3 is a central activator of all nitrogen catabolism which generates rather than utilizes nitrogenous precursors. Therefore, it is not too surprising that control of pathways generating and using nitrogenous precursors might share similarities on the one hand and exhibit distinct differences on the other. An analogous situation occurs with general amino acid control by the GCN proteins (Riego *et al.* 2002; Sosa *et al.* 2003; Hernandez *et al.* 2011). It is well known that the regulation of protein synthesis by the GCN proteins is mechanistically distinct from the NCR-sensitive regulation of Gln3 and its control of nitrogen catabolism even though Gln3 provides the nitrogen for that protein biosynthesis. Additionally, the two regulatory systems often collaborate to fine tune expression of some, but not all, nitrogen catabolic genes. By this reasoning, results obtained using Sch9 phosphorylation and Gln3-Myc^13^ localization as reporters may, as we saw throughout this work, reveal both distinct and overlapping functions of TorC1- and NCR-dependent regulation in the control of nitrogenous compound degradation and reuse of the products it generates in biosynthetic reactions.
